# Flexible Polyurethane Foams from Bio-Based Polyols: Prepolymer Synthesis and Characterization

**DOI:** 10.3390/polym15224423

**Published:** 2023-11-16

**Authors:** Simona Losio, Angelica Cifarelli, Adriano Vignali, Simona Tomaselli, Fabio Bertini

**Affiliations:** Institute for Chemical Sciences and Technologies “G. Natta” National Research Council, Via A. Corti 12, 20133 Milan, Italy; angelica.cifarelli@gmail.com (A.C.); adriano.vignali@scitec.cnr.it (A.V.); simona.tomaselli@scitec.cnr.it (S.T.); fabio.bertini@scitec.cnr.it (F.B.)

**Keywords:** bio-polyols, toluene diisocyanate (TDI), flexible polyurethane foams, prepolymer synthesis and characterization, NMR, thermal and mechanical properties

## Abstract

Bio-polyols (BPOs), characterized by a hydroxyl number up to around 90 mg KOH/g, narrow polydispersity index and relatively low molecular mass up to 2000 g/mol, were synthetized from partially and completely epoxidized soybean and linseed oils and caprylic acid or 3-phenyl butyric acid. These BPOs were used in the presence of toluene diisocyanate to produce polyurethane (PU) foams by using a quasi-prepolymer method involving a two-step reaction. A detailed structural investigation of the prepolymers from toluene diisocyanate and both BPOs and polypropylene glycol was conducted by SEC and solution NMR. The apparent density of the foams was in the range of 40–90 kg/m^3^, with higher values for foams from the aromatic acid. All the foams showed an open-cell structure with uniform and regular shape and uniform size. The specific Young’s moduli and compression deflection values suggest superior mechanical properties than the reference foams. The novel synthesized polyurethanes are excellent candidates to partially replace petroleum-based materials.

## 1. Introduction

Nowadays, growing ecological problems and the global political tendencies promote more sustainable chemistry and particularly the use of renewable resources to synthesize bio-based chemicals. The polyurethane (PU) industry is also looking into renewable raw materials to replace the major feedstocks which are derived from petroleum sources. PUs are obtained by the reaction of an oligomeric polyol (i.e., polyester or polyether polyol) and a diisocyanate. They represent a class of segmented copolymers composed of soft and hard segments. From a molecular perspective, polyether or polyester polyol usually represents the soft segment while the diisocyanate and chain extenders constitute the hard segments [1].

In the last few decades, the chemical industry has shown a strong interest in the use and production of bio-based rather than petroleum-based polyols. Among the large number of recently developed bio-based polyols, those synthetized from biomass such as vegetable oils [2,3,4,5,6] are of particular interest since they satisfy the requirements needed for the synthesis of PU foams. Recently, we prepared bio-polyols from epoxidized soybean (ESO) and linseed oils (ELO) and caprylic acid (CA) or 3-phenyl butyric acid (3PBA), using an environmentally friendly, solvent-free method in the presence of triethylamine (TEA) as the catalyst [7,8]. These bio-polyols were used to prepare flexible polyurethane foams with an open cell structure in combination with Tolonate^TM^, an aliphatic bio-based diisocyanate, to achieve a material with a reduced carbon footprint.

It is well known that there is a close relationship between the chemical structure and the polyol properties and the behavior and type of the corresponding foams. The rigidity of the foam depends on the number of hydroxyl groups present in the polyols. Polyols for soft PU foams generally contain low numbers of hydroxyl groups, around 5–100 mg KOH per g [2] and molecular weights from 1500 to 10,000 g/mol. There is great industrial interest in flexible foams for use in automotive, furniture, packaging, and bedding applications. Aromatic toluene diisocyanate (TDI) is usually used for producing flexible foams since it is more reactive compared to other isocyanates. Diisocyanates are frequently used in nonequivalent amounts over polyols for the polyurethane prepolymer synthesis. These linear, isocyanate-terminated oligomers are widely used as precursors of the corresponding polymers contributing to a wide variety of consumer applications such as coatings, paints, and household goods [9,10]. In PU technology, prepolymers are widely used because the final thermal and mechanical properties of the products can be determined and fine-tuned by the prepolymers [1]. During prepolymer synthesis, attention should be paid to the increasing viscosity and crosslink density of the system, which may often be the limiting factor in generating technically processible and commercially useful prepolymers.

Several studies have been conducted to determine the chemical structure of the polyurethane prepolymers by different techniques such as size exclusion chromatography (SEC) [11], NMR spectroscopy [12,13,14], and mass spectrometry (MS) [15]. Prepolymers are prepared by the reaction of linear and branched polyols with different isocyanates such as TDI [11], isophorone diisocyanate [16], and methylene bis(phenyl-isocyanate) [17]. A structural investigation of a prepolymer from TDI and trimethylolpropane was conducted as well by SEC, ^1^H NMR and MS [18].

In this study, we report the synthesis of flexible bio-based polyurethane foams from bio-polyols and TDI via prepolymer synthesis. PU prepolymers were synthesized in two steps: first, by reacting polypropylene glycol (PPG) with TDI, giving isocyanate-terminated intermediates; these were then reacted, in a second step, with different bio-polyols (from now on BPOs) from CA or 3PBA grafted to partially or completely epoxidized vegetable oils. The second reaction step is more complex than the first one, because it involves BPO, a multifunctional component with secondary −OH groups, which typically exhibits lower reactivity than primary hydroxyl groups. The prepolymers were accurately characterized by SEC and NMR spectroscopy. Solution NMR characterization of prepolymers prepared from commercial TDI (i.e., a mixture of 2,4- and 2,6-TDI) using deuterated dichloromethane as a solvent, instead of the common solvents for PU (acetone-d_6_, chloroform-d or DMSO-d_6_) is discussed. The assignment of the prepolymers protons, carbons and nitrogen were performed by a set of 1D and 2D NMR experiments (HSQC and HMBC). To the best of our knowledge, this is the first example of microstructural characterization by NMR of a prepolymer prepared in the presence of both PPG and vegetable oil-based polyols, whereas the isocyanate-terminated PU prepolymers reported in the literature come only from a polyol or PPG [12,13,14,16,18].

Then, the prepolymers so prepared were reacted with glycerol and water. The polyurethane foams were thus characterized in terms of morphology (cellular structure, interconnectivity), density, mechanical, and thermal properties and the ensuing results compared to those obtained from foams obtained exclusively from PPG in the absence of bio-polyols. Our studies revealed that: (i) PU foams from BPOs exhibit better performance than their PPG-based reference counterparts; (ii) among the BPOs, the best results in terms of the best reactivity, Young’s moduli, and compression deflection values were achieved with bio-polyol from ELO and 3PBA, due to the presence of aromatic moieties.

All these results evidence the importance of the development of prepolymers based on polyols from renewable sources in the synthesis of TDI-derived polyurethane, which may constitute a potential candidate for partially replacing petroleum-based polyurethanes.

## 2. Materials and Methods

### 2.1. Materials

The BPO syntheses were carried out on a double manifold Schlenk vacuum line under nitrogen atmosphere. Epoxidized linseed oil (99.0%) with approximately 6.4 epoxy group per triglyceride and *M*_n_ equal to 1182 g/mol, was supplied by Hallstar (Chicago, IL, USA) and used as received. Soybean oil (99.0%), diethyl ether (≥99.0%), sodium bicarbonate (≥99.0%), 1,4-diazabicyclo [2.2.2]octane (98%), toluene-2,4-diisocyanate (tech. 80%, remainder 2,6-diisocyanate), glycerol (≥99%), triethylamine (98%), and methanol were purchased from Fisher Scientific Company (Hampton, NH, USA). Caprylic acid (≥99%) and 3-phenyl butyric acid (99%) were purchased from Carlo Erba (Milan, Italy). Niax Silicone L-537 XF (Momentive, Tarrytown, NY, USA) was kindly supplied by Eigenmann & Veronelli SpA (Milan, Italy). Polypropylene glycol with *M*_n_ = 4600 g/mol evaluated from our SEC system and dibutyltin dilaurate (≥99.0%) were purchased from Merck KGaA (Darmstadt, Germany). Deuterated solvent, CD_2_Cl_2_, and hexamethyldisilane for NMR measurements were used as received from Merck KGaA (Darmstadt, Germany).

### 2.2. Epoxidation of Soybean Oil

The epoxidation of the soybean oil (SO) was conducted according to references [19,20] with an in situ formation of performic acid from formic acid (3 equiv/SO) and hydrogen peroxide (2.5 equiv/HCOOH). Briefly, in a two-necked flask connected to refrigeration, the peroxide solution was carefully added to the SO through a dropping funnel at ambient temperature, the reaction mixture was then reacted at 60 °C, overnight. The solution was neutralized with NaHCO_3_, and the epoxidized oil extracted with diethyl ether and then washed twice with water. Finally, the solvent was removed under vacuum thus obtaining the final product.

### 2.3. Synthesis of Bio-Polyols

BPOs were prepared according to our previous work [7]. Briefly, epoxidized oil (100 g) was placed in a three necked flask, equipped with a condenser. TEA (1% of epoxidized oil weight) was added to reaction flask and was stirred at 40 °C for 40 min. Under continuous stirring, organic acid was added to the reactor dropwise (1 drop/s).

### 2.4. Preparation of PU Foams via Prepolymer Synthesis

PU intermediates were synthesized in a closed vessel at atmospheric pressure under mechanical stirring. Quasi-prepolymers [21] were prepared by first reacting a large excess of TDI (free isocyanate content about 30 wt %) with PPG for 40 min in the presence of dibutyltin dilaurate (0.01 wt %) and silicone surfactant (0.5 wt %); afterwards, the isocyanate-terminated intermediate formed in the previous step was reacted with bio-polyol.

PU foams were prepared by mixing the PU prepolymer in a pre-heated container with catalyst, silicone surfactant, glycerol and diisocyanate, using a mechanical stirrer at 1400 rpm for 25 s. Distilled water (1.5 wt %) was used as blowing agent. An isocyanate index, that is, the excess of isocyanate over the theoretical amount for 1:1 reaction with all active hydrogen-bearing groups expressed in percentage terms, equal to 100 was reached. The expanded PU foams were crushed after foaming and then cured for 2 h in an oven at 60 °C. The chemical-physical characterization was performed on the core of the cylindrical samples where the cellular structure is homogeneous, and the edge effects are negligible.

### 2.5. Characterization Techniques

The hydroxyl number of the polyol (OH number, mg KOH/g polyol) was determined by the imidazole-catalyzed pyromellitic dianhydride method, according to ASTM D 4274-99 [22]. The acid value was determined according to IUPAC 2.201 standard using the indicator method [23].

^1^H-NMR spectroscopic analysis of the polyols was carried out on a Bruker (Billerica, MA, USA) Avance 500 MHz NMR spectrometer in CD_2_Cl_2_ at 25 °C. HMDS signal was used as internal reference. The area of the resonance belonging to a proton at 5.12 ppm was used as internal standard [24].

NMR prepolymer samples were prepared by dissolving 20 mg in 600 µL of CD_2_Cl_2_. Dilution of 15% was performed by adding fresh CH_2_Cl_2_ to the samples. NMR spectra were recorded at 25 and 35 °C with a Bruker (Billerica, MA, USA) Avance II and Bruker (Billerica, MA, USA) DMX instruments operating at 500 MHz and 600 MHz, respectively. Two-dimensional ^1^H–^1^H Clean-TOCSY, COSY, NOESY, ^13^C HSQC, HMBC experiments were performed, using established methods [25,26]. TOCSY mixing time was 100 ms with a delay of 91 µs, NOESY experiment was recorded with 400 ms mixing time. Chemical shifts were measured relative to residual CH_2_Cl_2_. ^15^N-HSQC was acquired with 512 scans. Spectra were processed and analyzed with the software Topspin 4.05.

Size exclusion chromatography (SEC) analyses were performed on a Waters (Milford, MA, USA) GPCV2000 system, using THF as mobile phase, at 35 °C with a 0.6 mL/min flow. Sample concentration was set at 2 mg/mL and injection volume at 150 µL. The SEC system was calibrated using polystyrene standards.

FTIR spectra were recorded using a PerkinElmer (Waltham, MA, USA) Spectrum Two spectrometer from 4000 to 550 cm^−1^ with a wavenumber resolution of 4 cm^−1^ in total attenuated reflectance (ATR) mode.

Thermogravimetric analysis (TGA) was performed on a PerkinElmer (Waltham, MA, USA) TGA 7 instrument at a scan rate of 10 °C/min under nitrogen atmosphere. TGA and derivative thermogravimetry (DTG) curves were recorded from 50 up to 750 °C.

The viscosity of the polyol was measured using a TA Instruments (New Castle, DE, USA) AR 2000 dynamic stress rheometer at 20 °C with a flow test in the shear rate range between 0.01 and 1000 s^−1^. The rheometer was equipped with a plate and steel cone (diameter of 20 mm, angle of 0.5 ° and truncation gap of 16 µm).

The cream time and free rise time of PU foams were evaluated according to the standard “cup-test” in ASTM D7487-13E1 using a digital timer. Each test was conducted repeatedly at least five times to minimize experimental error [27].

The apparent density of foam samples was measured according to EN ISO 845:2006 [28].

The morphology of foams was investigated using a Thermo Fisher Scientific (Waltham, MA, USA) Phenom XL G2 scanning electron microscope (SEM) operating at 10 kV. Before the observations, the samples were blade cut from the core of the foams. The Phenom (Eindhoven, The Netherland) PoroMetric 1.1.2.0 software was used to analyze the pore sizes. For each sample, the average dimension of pores was obtained from approximately 100 different measurements.

Mechanical properties of the foams were analyzed through compression force deflection tests performed by a Zwick-Roell (Ulm, Germany) Z010 mechanical testing machine with a load cell of 2.5 kN. Cylindrical-shaped specimens (height of 25 mm and diameter of 50 mm) were firstly pre-compressed twice to 75% of their initial height at 250 mm/min. After a rest of 6 min, the height of each specimen was determined by applying a load of 140 Pa. Finally, the specimens were compressed to 50% of their height at 50 mm/min and the final load after 60 s was recorded. Reported data were averaged on three tests per sample.

## 3. Results

### 3.1. Synthesis and Proprieties of Bio-Polyols

**Scheme 1:**
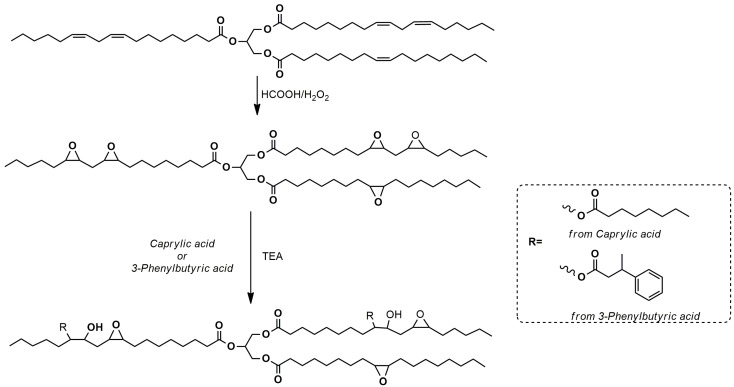
Schematic representation of the synthesis of bio-polyols.

Renewable BPOs with several functionalization degrees were prepared from two different no-food vegetable oils, linseed and soybean oil, using a solvent-free method [7], in which no purification or separation steps were required (Scheme 1).

For BPOs from linseed oil, a commercial epoxidized linseed oil (ELO) was used, while for those from soybean oil, one partially and one completely epoxidized soybean oil (p-ESO and ESO, respectively) sample was prepared from a commercial soybean oil to evaluate the effect of the presence of residual double bonds on the final properties of the ensuing polyurethane foams. p-ESO and ESO were obtained from the reaction of soybean oil with formic acid and hydrogen peroxide overnight at 60 °C (Scheme 1). Two oil/peracid ratios were used to tune the epoxidation degree (Table 1).

**Figure 1:**
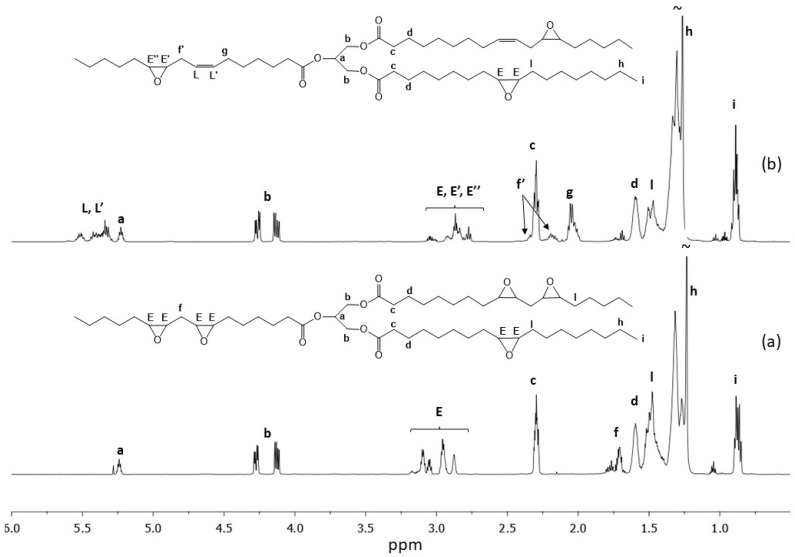
^1^H NMR spectra of fully epoxidized (**a**) and partially epoxidized soybean oil (**b**), respectively. Uppercase letters refer to protons of epoxy groups or double bonds.

In Figure 1, the ^1^H NMR spectra of partially and fully epoxidized soybean oils are reported.

For ESO (Figure 1a), all the double bonds were converted into epoxy groups as confirmed by the absence of the protons related to olefinic *L*, *L*′ proton atoms in the region from 5.48 to 5.24 ppm [29], and by the presence of resonances in the regions from 2.82 to 2.97 ppm and 3.00 to 3.17 ppm, related to protons of epoxy groups (E). The ^1^H NMR spectrum of p-ESO (Figure 1b) shows the simultaneous presence of resonances belonging to epoxy groups (protons *E*, *E*′ and *E*″) at 3.00–2.87 ppm and of resonances related to double bond in the region spanning from 5.48 to 5.24 ppm, (protons *L*, *L*′). Moreover, there is the appearance of two signals from 2.15 to 2.25 ppm and at 2.36, overlapped with the resonance at 2.34 (protons *c*), due to methylene protons between an epoxy and an olefinic group (protons *f*′). The presence of a resonance at 2.08 ppm ascribed to *g* protons, due to allylic methylenes, further confirms the partial epoxidization.

^1^H NMR analysis assessed an estimated epoxy group per triglyceride of 2.8, 5.1 and 6.4 for p-ESO, ESO, and epoxidized linseed oil (ELO), respectively, (Figure S1).

For the synthesis of the bio-based polyols, caprylic and 3-phenylbutyric acid, as the organic acids, respectively, were used to analyze the effect of an aliphatic short linear chain or the presence of an aromatic moiety on PU formulation and properties.

It is worth noting that the hydroxyl functionality, viscosity, residual double bonds, or epoxide in the bio-polyols backbone are key issues for the synthesis of PU foams. With a complete conversion of the epoxide group per triglyceride, a maximum total number of OH groups was estimated around 5–6. However, with these high functionalities, rigid foams were expected [21,30,31]. Flexible foams require low crosslink density which can be obtained by using polyols with either low OH functionalities (in the range between 2 and 3) or molecular weights up to 1500 g/mol. Thus, polyols with those features were prepared and are reported in Table 2. Highest viscosity values were observed for both bio-polyols from 3-phenylbutyric acid (Table 2) due to the presence of the aromatic ring that increases the rigidity of the side-chain and the friction resistance [32].

**Scheme 2:**
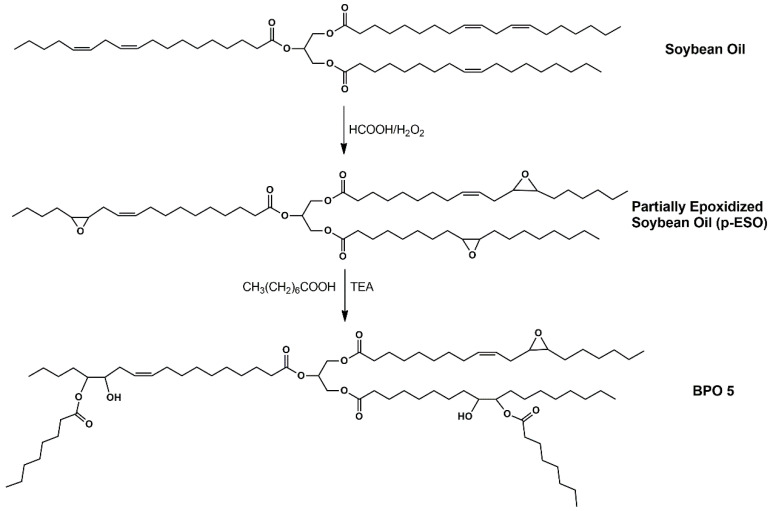
Schematic representation of the synthesis of BPO 5 in Table 1.

When caprylic acid is grafted on the epoxidized oil backbone, the viscosity dramatically decreases and as expected, the polyol from p-ESO and caprylic acid (BPO 5 in Table 1) showed the lowest viscosity due to its structural properties (Scheme 2). No bio-polyol was prepared by reacting p-ESO and 3-phenyl-butyrric acid since, as already mentioned, the presence of the aromatic moieties greatly increases the viscosity [32].

**Figure 2:**
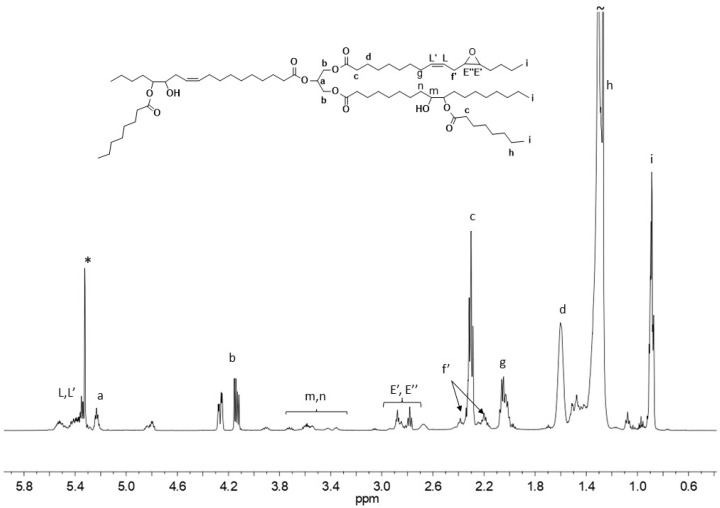
^1^H NMR spectrum of bio-polyol from partially epoxidized soybean oil and caprylic acid (BPO 5 in Table 2). (NMR solvent is starred). Uppercase letters refer to protons of residual epoxy group or double bonds.

The ^1^H NMR spectrum of bio-polyol from p-ESO confirmed the simultaneous presence of resonances related to the double bond in the region spanning from 5.48 to 5.24 ppm, (proton *L*), between 2.82 and 3.17 related to protons *E* (epoxide group) and between 3.20 and 3.80 ppm due to methylene and methine groups in the beta and alpha positions to the hydroxyl groups (Figure 2) [33,34]. A residual double bond functionality of 2 was determined for this bio-polyol.

**Figure 3:**
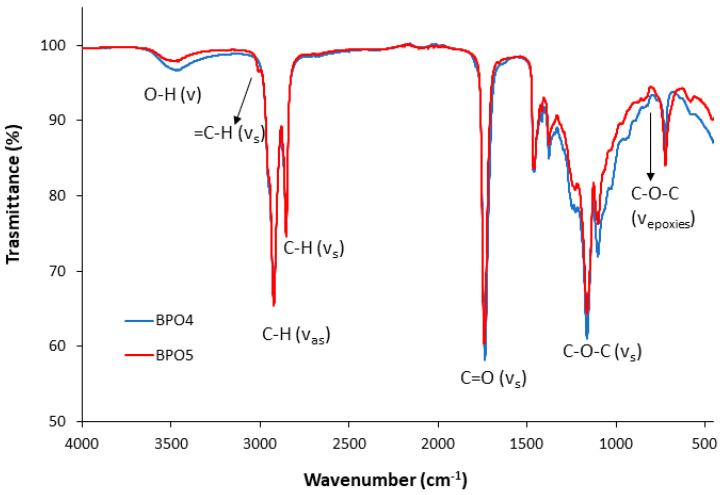
FT-IR spectra of the bio-polyols from ESO or p-ESO and CA (BPO 4 and 5 in Table 2).

The FTIR spectra of the bio-polyols from p-ESO or ESO and caprylic acid are presented in Figure 3. They both show between 3600 and 3200 cm^−1^ the broad absorption peak characteristic for stretching vibrations of OH groups; at 1732 cm^−1^, the C=O stretching vibration of esters and at ~2924 and 2854 cm^−1^ the –CH_2_ symmetric and asymmetric stretching [35].

The oxirane ring vibration peak at 824 cm^−1^ of epoxidized vegetable oils had a weak presence, according to NMR analysis. Moreover, the polyol from partially epoxidized oil showed the presence of a small absorption peak characteristic for C-H stretching ascribed to residual double bond around 2990 cm^−1^ [36].

The FT-IR spectra of BPOs from the aromatic acid (BPO 1 and 2), not reported, displayed the typical absorption peaks of monosubstituted aromatic groups from 3-phenyl butyric acid [7].

**Figure 4:**
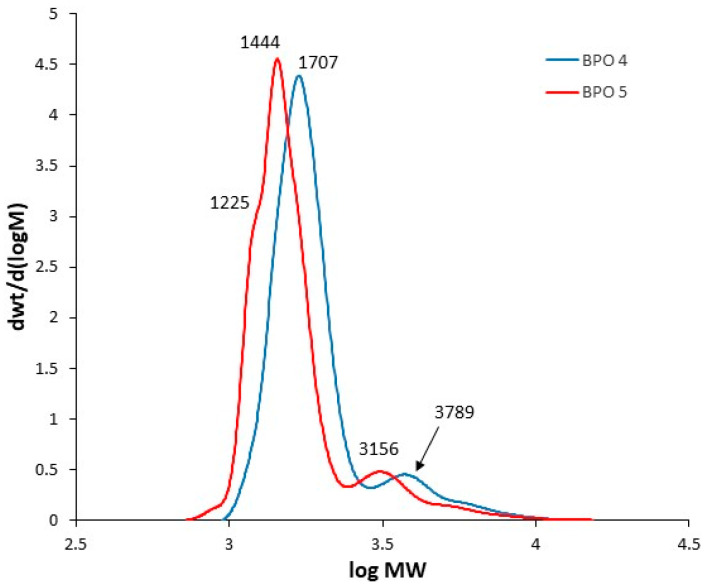
SEC chromatograms of bio-polyols from ESO or p-ESO and CA (BPO 4 and 5 in Table 2).

The SEC curves as a function of molecular weight (MW) for the bio-polyols from p-ESO or ESO and CA (Figure 4) showed two fractions, the main one at lower molecular weight whose molecular weight depends on the functionalization degree of the bio-polyol and a second one at a higher molecular weight ranging from 3100 to 3800 g/mol, corresponding to the oligomeric fraction with an ester functionalization degree of between two and three per oil molecule.

**Figure 5:**
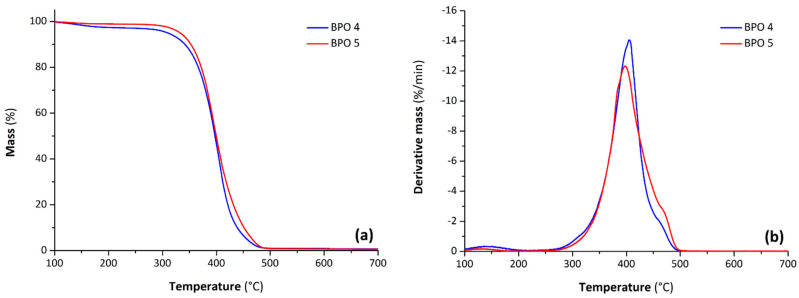
TGA (**a**) and DTG (**b**) curves of selected bio-polyols, BPO 4 and 5 in Table 2.

The polyols were characterized by TGA: the thermograms of bio-polyols from partially and completely epoxidized soybean oils are depicted in Figure 5a,b, respectively. The thermal analysis proved that the use of p-ESO in the preparation of polyol does not produce significant differences from the bio-polyol from ESO. These samples, synthesized from CA, showed good thermal stability under nitrogen, as highlighted by a temperature of degradation at 5% mass loss of 310 °C for BPO 4 and 332 °C for BPO 5. Both samples exhibited a first degradation event with a mass loss of 2–4% ascribed to the presence of a small amount of unreacted acid at low temperature (see faint DTG peak centered at about 140 °C). The principal degradation stage of the bio-polyols from ESO and p-ESO occurred between 300 and 500 °C with a corresponding DTG peak at about 400 °C. Moreover, both the polyols presented a residual mass at 700 °C in nitrogen lower than 1%.

### 3.2. Prepolymer Synthesis

**Scheme 3:**
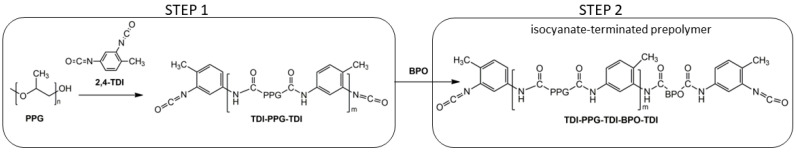
Schematic representation of synthetic pathway to obtain prepolymers.

For the synthesis of polyurethane foams, a PU quasi-prepolymer was synthesized by a two-step reaction (Scheme 3) in the presence of an excess of TDI (total index 200). The one-shot approach, by-passing prepolymer synthesis, was excluded since preliminary tests showed that it leads to easy cracking and crumbling foams. Moreover, a prepolymer leads to better compatibility between additional polyurethane formulation components, since the reacted systems provide lower surface tension to other chemicals within the solubility parameter range.

The first step (Scheme 3) involves the reaction between the polypropylene glycol (PPG) and a large excess of diisocyanate, giving isocyanate-terminated intermediates [TDI-(PPG-TDI)_m_]. For short reaction times, the majority of the prepolymer consists of a single adduct, TDI-PPG-TDI, with a molecular weight equal to the molecular weight of PPG plus the molecular weight of two diisocyanate units capping the polyol, while with an increased reaction time, longer oligomers (i.e., sequences of alternating TDI and PPG) can be observed.

In the second step, BPO reacts with the PPG-TDI adduct for 30 min at 40 °C, giving an isocyanate-terminated quasi-prepolymer. This step is more complex than the first one, because it involves the bio-polyols that are reactants with secondary −OH groups and with functionality ≥ 2. The number of branches of the quasi-prepolymer is strictly related to the functionality of the raw materials used to prepare the isocyanate-terminated prepolymer.

At the end of the second step, the products should be end-capped polyols (TDI-BPO-TDI) with a molecular weight corresponding to the molecular weight of bio-polyol plus twice the molecular weight of the diisocyanate, and species with higher molecular weights, which consist of isocyanate-terminated prepolymer TDI-(PPG-TDI)_m_-BPO-TDI with *m* corresponding to that obtained in the first step. In our conditions, the prepolymer should be TDI-PPG-TDI-BPO-TDI.

**Figure 6:**
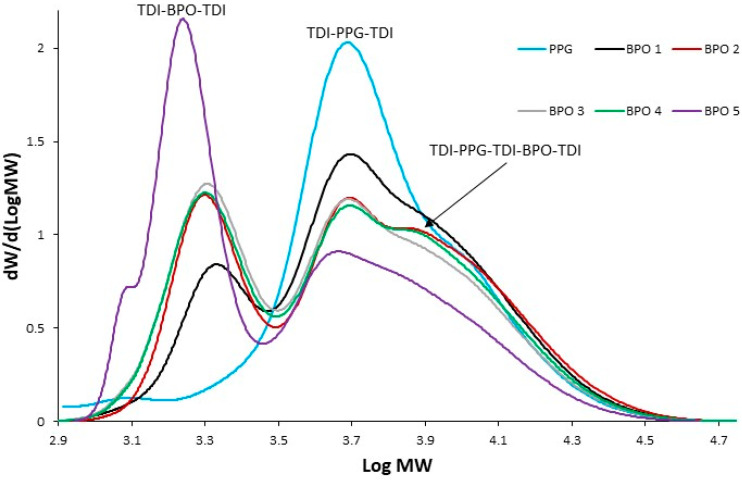
SEC chromatograms of prepolymers from all bio-polyols and from PPG.

To have an insight into the synthesis of the prepolymers, the final products of each of the two steps were stirred with methanol for 30 min to cap any unreacted isocyanate groups and afterwards characterized by SEC and ^1^H NMR. In Figure 6, SEC chromatograms of prepolymers from all bio-polyols are depicted and compared to that from PPG, obtained in the first step. Different products can be observed: (i) end-capped polyols, TDI-BPO-TDI, with lower molecular weight, (ii) end-capped PPG, TDI-PPG-TDI, molecular weight about 5000 g/mol, and (iii) species with higher molecular weight, corresponding to the TDI-PPG-TDI-BPO-TDI alternating structure, with linear or branched structures (e.g., for bio-polyols with functionality > 2).

It is worth noting, no significant differences were observed concerning the reactivity of the different bio-polyols with diisocyanate from SEC analysis. Moreover, BPO 5 (low OH functionality = 2), the vegetable oil lacking −OH groups in some portions of the backbone due to the presence of unreacted double bonds, resulted in being partially unreacted as shown in the SEC chromatogram of (see also Figure 4).

Preferably, the number of branches of the isocyanate prepolymer and the crosslink density, i.e., the number of attachments between chains of polymers, should be kept low to control the final viscosity of the PU intermediate.

A prepolymer from bio-polyols and TDI in the absence of PPG was prepared for comparison to better understand how the reaction proceeds. It is worth noting that, in the absence of PPG, the reaction medium, made of bio-polyol and TDI in the presence of dibutyltin dilaurate as catalyst, reached the crosslinking point after 20 min at 40 °C, giving a semi-solid product. According to the Flory–Stockmayer theory, the higher the functionality of the monomers, the lower the conversion at the gel point, and consequently, the lower the gel time will become [37,38].

From all these considerations, it is possible to control the reaction between bio-polyols and TDI, (Scheme 3), by introducing PPG, with linear long chains. It is reasonable to suppose that the end-capped PPG decreases the overall viscosity of the system, also partially reacting with the bio-polyol leading to linear copolymers limiting the growth of hyper-branched chains in the prepolymer; that is, the uncontrolled reaction between multiple end-capped BPO units.

**Figure 7:**
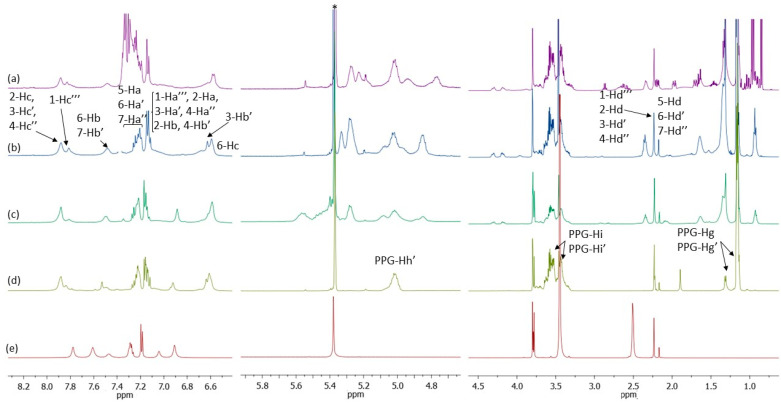
Different spectral regions of ^1^H-NMR spectra of prepolymers obtained from selected bio-polyols: BPO 2 (**a**), BPO 4 (**b**), BPO 5 (**c**), and PPG and quenched by MeOH. ^1^H-NMR spectra of prepolymers from PPG (**d**) and TDI (**e**) quenched by MeOH are reported as reference. C_2_D_2_Cl_2_ signal is starred.

**Scheme 4:**
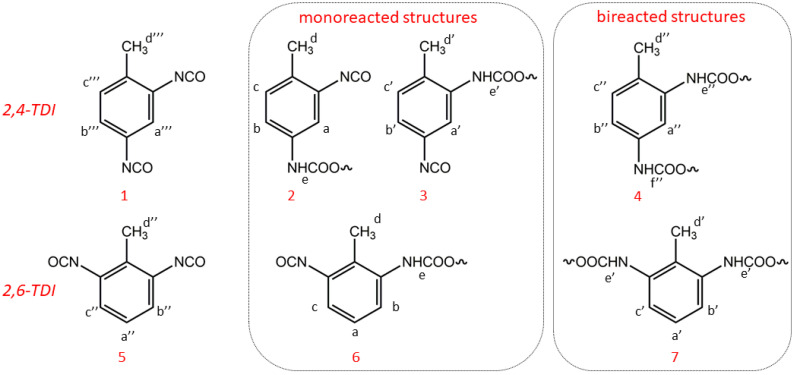
Chemical structures of the products obtained from TDI and their ^1^H-NMR assignment.

**Scheme 5:**
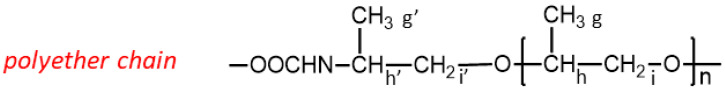
Chemical structure of the products obtained from the reaction of PPG and TDI and their ^1^H-NMR assignment.

The ^1^H-NMR spectra (Figure 7) of the prepolymers, quenched by methanol, validate the successful reaction between polyols (PPG and bio-polyols) and TDI. The complete assignment of the resonances ascribed to the structure coming from TDI (Scheme 4) and PPG (Scheme 5) have been reported; where no assignments are indicated, the corresponding resonances can be attributed to the BPOs. Moreover, the ^1^H NMR spectra of PPG-prepolymer and TDI are reported as reference (Figure 7d,e).

The ^1^H-NMR spectra were analyzed and compared to those reported by Pegoraro et al. for prepolymers prepared from toluene 2,4-diisocyanate and toluene 2,6-diisocyanate using polypropylene glycol and acetone-d_6_ as solvent [11]. Indeed, the complexity of the spectra acquired from samples elicited using commercial TDI where two positional isomers, toluene 2,4-diisocyanate (2,4-TDI) and toluene 2,6-diisocyanate (2,6-TDI) are present, usually in the ratio 80:20, is well known. Three structures with 2,4-TDI and two with 2,6-TDI, as reported in Scheme 4, are expected by reacting with TDI. When 2,4-TDI is used, species can be formed (1) by reaction of the more reactive NCO in the para position to the methyl group, (2) from the less reactive NCO in ortho position to the methyl group, and (3) from a biurethane structure. With 2,6-TDI, due to the symmetry of the system, both monoreacted or bireacted structures can be expected.

Thus, the more reactive para-NCO group preferentially reacts with the OH groups of PPG or bio-polyol to form an adduct that is terminated with the less reactive ortho-NCO groups if the reaction is carried with an excess of NCO groups. The ^1^H NMR of prepolymers should show that a compound terminated with the less reactive ortho-NCO groups represented the main form of the urethanes in the polyol-TDI.

It is evident that the use of the two different polyols makes the NMR spectrum pattern more complex due to all the different expected chemical structures. The spectra on the left refer to the region spanning from 6.0 to 8.4 ppm, corresponding to those resonances arising entirely from aromatic protons of TDI and, for the prepolymer from BPO 2, at 7.2 ppm from proton atoms coming from 3-phenyl butyric acid. Resonances are labelled according to Scheme 4 and Scheme 5, where the number refers to the structure and the letter to the proton (i.e., protons 6-Ha are methyl protons of structure 6 of monoreacted structure from 2,6-TDI).

The complex group of signals in the 4.7–5.4 ppm range is due to the methine protons involved in the urethane linkages in bio-polyols confirming the reaction between polyol and TDI and the formation of urethane linkages. In detail, by analyzing spectrum (b) from PPG, and according to the literature [11], it is evident that the signal at 5.02 ppm can be safely assigned to the PPG methine protons *h*’ involved in the urethane linkages (Scheme 5). Resonance centered at 5.27 ppm corresponds to methine proton of the glycerol structure of bio-polyols.

Looking at the spectra on the right, the two multiples centered at 4.29 and 4.18 ppm, absent in spectrum (b), are related to methylene protons of the glycerol structure of polyols while resonances in the 3.2–3.5 ppm range are assigned according to the literature to the methine *h* belonging to the internal units and to the methylene protons *i* and *i*′ of PPG, as well as to bio-polyols for comparison with the spectra of the starting bio-polyols.

Resonances at 2.10 and 2.22 ppm were assigned to methyl protons in MeOH-capped TDI, while those centered at 2.50 ppm to TDI methyl protons.

The characteristic resonances of the methyl proton atoms in the polyol are located at 0.8 ppm for bio-polyols and in the region spanning from 1.14 to 1.30 ppm for PPG; in detail, these two resonances are due to the methyl protons belonging to the internal portion of the polyether chain (more abundant) and to the terminal units involved in the urethane linkages, respectively.

**Figure 8:**
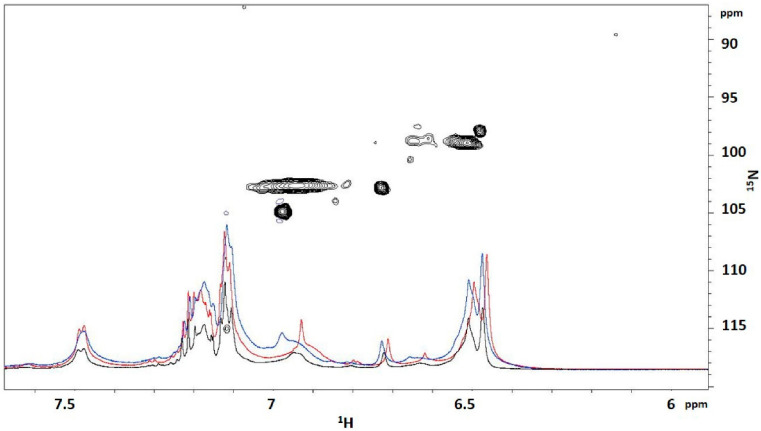
^15^N-HSQC of prepolymer from BPO 4 and corresponding region of 1D spectrum acquired at 25 °C (blue), 35 °C (red) and on the diluted sample (black).

To confirm the presence of the urethane bonds, 1D experiments were carried out at two different temperatures, 25 and 35 °C, allowing the identification of NH resonances. By increasing the temperature or by diluting the sample, NH resonances experience an upfield shift because of the hydrogen bonds progressive breaking, as observed in the 1D overlapping in Figure 8. To definitely assign urethane resonances, the ^15^N-HSQC spectra of the prepolymer from BPO 4 were acquired in natural abundance at 25 °C (Figure 8). In this spectrum, the expected NH resonances (structures 2, 3, 4, 6 and 7 of Scheme 4) were clearly visible between 6.4 and 7.1 ppm. The upfield shift urethane resonances appeared in a different spectral range, within the region around 8.5, as reported in the literature [11]. This is due to the absence of H bonds with the solvent since spectra are acquired in CD_2_Cl_2_. Further experiments are necessary to univocally assign these resonances.

**Figure 9:**
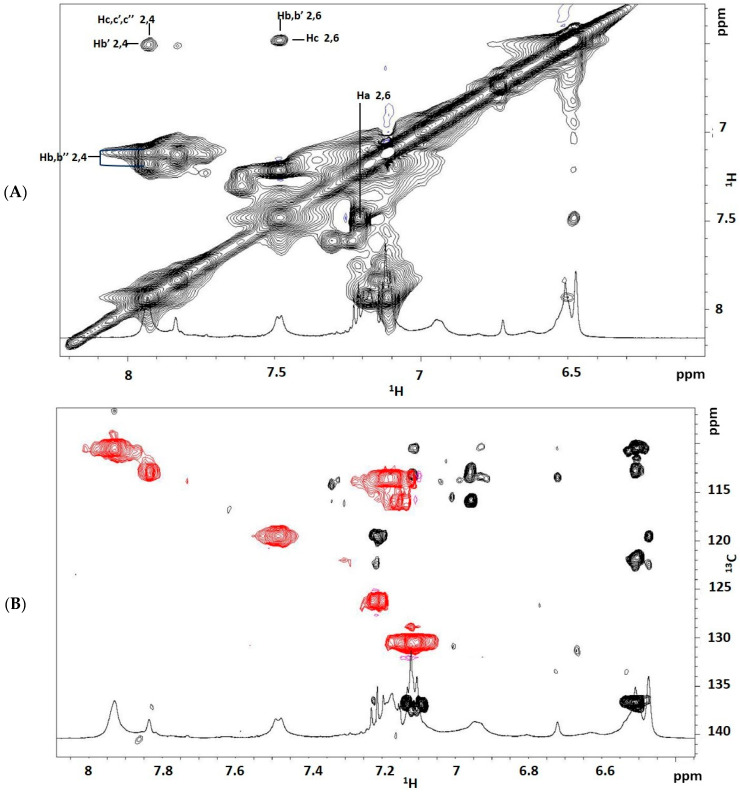
(**A**) Clean-TOCSY selected region of prepolymer from BPO 4. Resonance assignment is reported according to nomenclature reported in Table 3. (**B**) ^13^C HMBC (black) and ^13^C-HSQC (red) of prepolymer from BPO 4. One-dimensional spectra are reported for clarity reasons.

^13^C HMBC, ^13^C-HSQC, clean-TOCSY and COSY spectra were acquired to assign the aromatic protons of the prepolymers obtained from toluene 2,4-diisocyanate and toluene 2,6-diisocyanate from now on indicated as reported in Scheme 4. In Figure 9, the TOCSY aromatic region is reported which enabled the proton assignment (Figure 9A), and the overlapping of ^13^C HMBC and ^13^C-HSQC (Figure 9B) which enabled the carbon assignment.

In Table 3, all the assigned aromatic resonances of the most abundant species are reported.

### 3.3. PU Foam Development and Formulation Optimization

The formulations of bio-based polyurethane foams are reported in Table 4 and labelled with the number of the corresponding bio-polyol from Table 2. For each formulation, a ratio PPG:bio-polyol equal to 1:1 was chosen; a chemical blowing agent (distilled water) was used, and its content kept constant at 2.6 parts per hundred parts (pphp). As blowing catalyst, 1,4-diazabicyclo [2.2.2]octane (DABCO) was tested for all the formulations and a constant amount of dibutyltin dilaurate, DBT, (0.1 pphp) was fixed. Glycerol, 6 wt/wt %, was used to enhance the bio-polyol miscibility with water and the overall reactivity and properties. TDI-1W was prepared from BPO 1 with a double content of water. TDI-3.2P was obtained doubling the amount of BPO 3 (PPG/bio-polyol ratio 1/2), thus achieving the largest content of sustainable material (47 wt %) with respect to other bio-based foams with an average amount of 25 wt %. For the sake of comparison, TDI-ref and TDI-refW were obtained exclusively from PPG in the absence of bio-polyols; moreover, for TDI-refW, a double amount of water was added.

After the curing reaction of all formulations, the absorption of −OH at 3510 cm^−1^ and −NCO stretching vibration band at 2260 cm^−1^ were not present in the FTIR spectra (see Figures S2–S6) of all foams listed in Table 4, thus suggesting that isocyanate and hydroxyl groups are completely reacted in the reaction conditions.

All the developed prepolymers showed a good miscibility with the other components of the formulation. The high viscosity of bio-polyol 1 (470.8 Pa·s, see Table 2) did not affect the preparation of the foams, without hindering the miscibility and compatibility with PPG. However, the TDI-2 foam was characterized by some yellow-colored striations, probably due to poor homogeneity and miscibility of components. Thus, a too rigid (85 kPa 50% compression deflection value) and quite inhomogeneous foam with high density was produced. A less relevant problem connected with the miscibility of components was also in TDI-4.

The existence of a sharply defined foaming start time, rising time and gel point is a peculiar characteristic of such polyurethane systems. The cream time and the free rise time were recorded during the foaming process and are listed in Table 5.

Summarizing, the rising was very fast for all foams (up to 45 s). TDI-1 and TDI-1W foams showed the best reactivity (25 and 18 s, respectively) probably due to the peculiar structure of the bio-polyol rich in aromatic moieties. These results seem to suggest that a better compatibility with the isocyanate can be achieved with this BPO. Moreover, in TDI-1W formulation, where double the amount of water was added as blowing agent, the exothermicity of the reaction was well controlled and, despite the short cream time, thorough mixing was achieved.

As observed in our previous study [7], the relatively high viscosity of the bio-polyols facilitates the formation of the framework of the urethane group in the gel reaction stage between the bio-polyols and isocyanate, resulting in a dense structure. The apparent density of the foams was in the range of 40–90 kg/m^3^.

**Figure 10:**
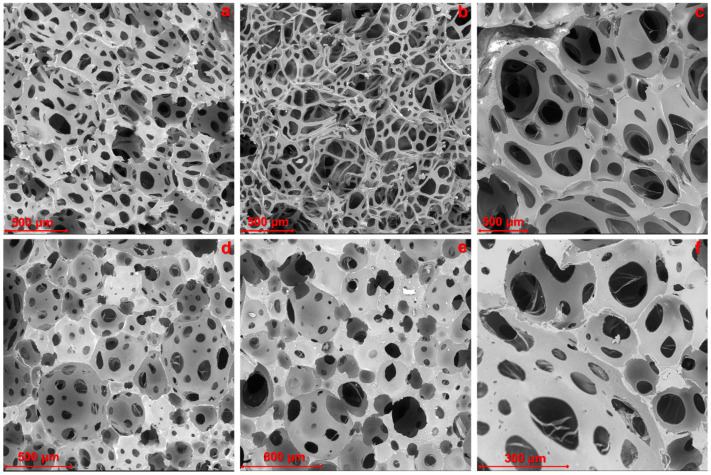
SEM micrographs of the selected PU foams: (**a**) TDI-1, (**b**) TDI-1W, (**c**) TDI-2, (**d**) TDI-3, (**e**) TDI-3.2P and (**f**) TDI-4.

The cells’ cross-section size and surface were determined based on SEM images. The average pore sizes with standard deviations for the different systems are reported in Table 6. The foam cells had an almost regular shape, indicating the quasi-isotropic growth of the bubble during the foaming process. In the case of samples with double the amount of water, the shape of the cells was less regular. This result may be attributed to high water content inducing a fast-blowing reaction with the isocyanate molecules. The pore size of foams was relatively uniform and presented a variability from 23 to 46% for the samples TDI-5 and TDI-ref, respectively. Recently, the authors reported on the preparation and characterization of formulations with some of the selected bio-polyols and Tolonate^TM^, a partially bio-based aliphatic isocyanate [7]. By comparing the dimensions of the cavities and cells from TDI with those obtained from Tolonate^TM^, it was evident that smaller cells, as well as thinner struts formed between interconnecting pores, could be observed in TDI-based formulations. It is probably due to the higher reactivity of the aromatic isocyanate leading to fast crosslinking of the structure. Figure 10, Figure S7 and Figure S8 show micrographs of the cross-section surface of selected foams.

The addition of bio-polyols based on ELO, regardless of the substituent groups, decreased the pore size; in particular, the foam TDI-3, based on ELO-CA, exhibited the lowest average pore size (54 µm). The presence of a double amount of the same bio-polyol in the sample TDI-3.2P resulted in a more than doubled pore size compared to TDI-3 (see Table 5). For TDI-5 from a partially epoxidized bio-polyol, a larger size of pores could be observed, indicating that the epoxidation degree of the bio-polyol has a great effect on the strut thickness.

The cell morphology of TDI-1W foam obtained by increasing the water content resulted in a very well-developed cavity structure. The average pore size increased and the bulk density decreased with increasing water content. This phenomenon was equally evident in the reference foams (TDI-ref and TDI-refW).

**Figure 11:**
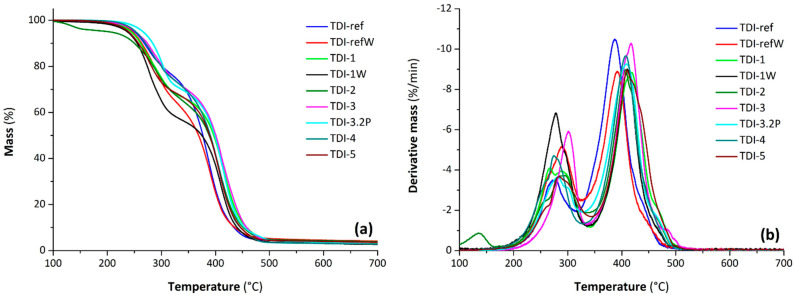
Thermogravimetric analysis (**a**) and derivative thermogravimetry (**b**) curves of PU foams.

The TGA and DTG curves of the PU foams are shown in Figure 11.

Generally, the thermograms of all samples presented a two-stage thermal decomposition under an inert atmosphere (Figure 11a): (i) the first mass-loss stage, recorded in the range from 200 to 320 °C with a mass loss between 20 and 40% (depending on the sample), is related to the dissociation of the urethane bonds [39]; (ii) the second stage occurred in the range from 320 to 500 °C and is attributed to the decomposition of polyol/bio-polyol components [40]. It is worth noting that at the second decomposition stage, the reference foam degrades faster than the foams obtained from bio-polyols, as reported in our previous work for the foams based on Tolonate^TM^ and the same polyol/bio-polyols [6]; in detail, the mass residue at 400 °C for the reference foams was close to 26%, while for the bio-based foams, it lay between 37 and 52%. TGA experimental data including the temperature at which the initial 5% mass loss occurs (*T*_5%_) and the temperatures of maximum rate of mass loss (*T*_max,1_ and *T*_max,2_ related to the first and second decomposition stage, respectively) determined by main DTG peaks are summarized in Table 6.

Regarding the *T*_5%_, the values ranged from 237 to 266 °C, except for TDI-2 where the partial presence of unreacted acid led to a mass loss highlighted by a DTG peak centered at 135 °C. The foams prepared with a double amount of water (TDI-1W and TDI-refW) exhibited a thermal stability comparable with the corresponding foams (TDI-1 and TDI-ref, respectively). The presence of a double content of bio-polyol in TDI-3.2P slightly reduced the characteristic degradation temperatures compared to TDI-3; the addition of the partially epoxidized bio-polyol in TDI-5 improved the thermal stability with respect to TDI-4.

**Figure 12:**
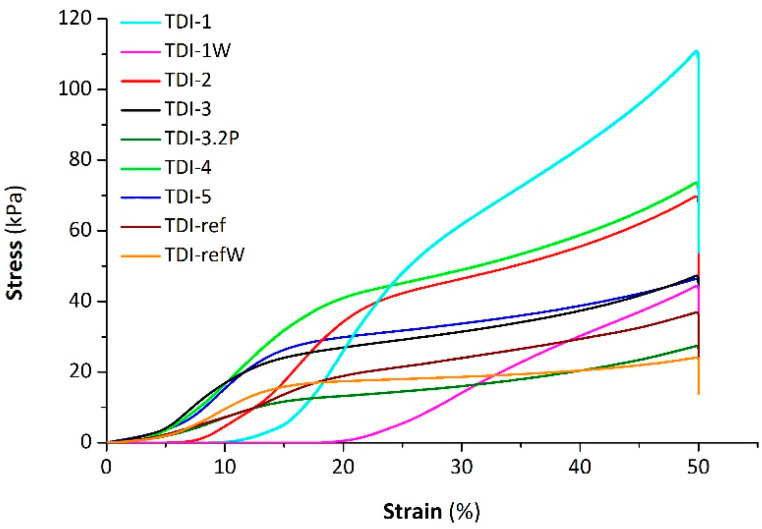
Stress–strain curves of PU foams.

The compressive stress–strain curves of the investigated samples in Figure 12 show the typical behavior of polyurethane foams: initial linear part, depending on the elastic deformation of the structure, followed by yield and constant enhancement of stress due to partial densification of cells as strain increases.

**Figure 13:**
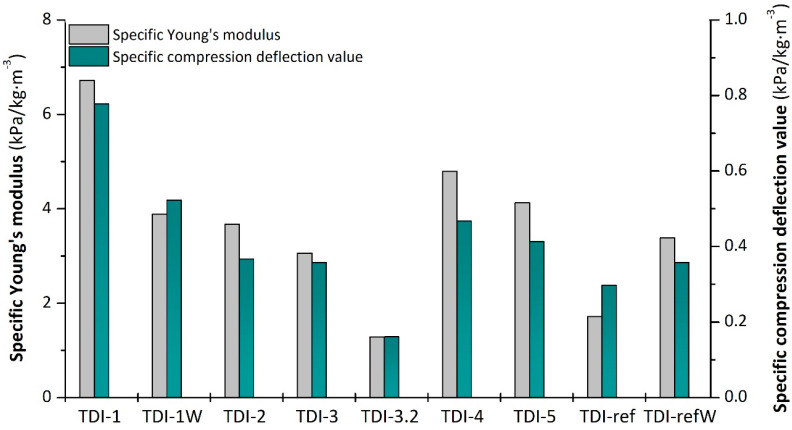
Specific mechanical properties of PU foams.

The mean values of Young’s modulus (*E*) and the compression deflection value (*CDV*), calculated as the ratio between the final load and the cross-section area of the sample at 50% of strain, are listed in Table 6. The compressive mechanical properties of the foams prepared with bio-polyols were higher than those of the reference foam. In particular, the presence of polyol based on ELO and 3PBA in TDI-1 caused a greater increase (respectively, 280 and 150%) in the Young’s modulus and compressive deflection value in comparison to the reference foam. This result is strongly correlated to the difference in terms of cavity size between TDI-ref and bio-based foam. Indeed, Gibson et al. demonstrated that the compression strength of ductile cellular materials is inversely proportional to their cell size [41,42]. Analogously, the bio-based foam containing the double content of water, i.e., TDI-1W, exhibited an enhancement of compressive properties compared to the respective reference foam (TDI-refW). Moreover, the compression properties of the foams obtained adding partially or totally ESO and CA, respectively, TDI-5 and TDI-4, were higher for the latter foam because of smaller pore size and higher density. As regards TDI-3.2P, mixing a double amount of bio-polyol caused an enhancement in pore size and a 50% decrease in modulus and compressive strength with respect to TDI-3, despite a similar density. The investigated foams differ in apparent density; thus their mechanical properties can be better compared by normalizing with respect to apparent density. The specific Young’s moduli and specific compression deflection values of all the analyzed samples are depicted in Figure 13 and confirm the trends so far observed.

In general, the bio-based foams showed superior mechanical properties than the reference foams. This interesting result confirmed the possibility of replacing polyurethane foams from fossil resources with bio-based foams endowed with improved mechanical performance.

## 4. Conclusions

Bio-polyols from partially or completely epoxidized soybean and linseed oil with narrow polydispersity indices and molecular mass up to 2000 g/mol were used to prepare flexible polyurethane foams in combination with commercial TDI, by the prepolymer method.

These polyurethane foams, with an apparent density in the range of 40–90 kg/m^3^, show Young’s moduli and compression deflection values that were usually higher than those of the reference foam, with the best results achieved with bio-polyol from ELO and 3PBA. This bio-based formulation showed the best reactivity in terms of free rise time, probably due to the peculiar structure of the bio-polyol rich in aromatic moieties.

The use of bio-polyols in the preparation of PU foams paves the way towards an extensive use of bio-based raw materials with tunable functionalities, e.g., unsaturated moieties, aliphatic or aromatic side-chains. Such bio-polyols may lead to novel advanced foams with improved ultimate properties.

## Figures and Tables

**Table 1:** Synthesis and characterization of epoxidized oils from soybean oil.

	SO/HCOOH (eq)	HCOOH/H_2_O_2_ (eq)	Epoxide Functionality ^1^	Double Bonds Functionality ^1^	*M*_n_ ^2^ (g/mol)	Ð ^2^
p-ESO	2:1	1:1	2.8	2.0	1169	1.01
ESO	1:3	1:2.5	5.1	0	1196	1.01

^1^ Determined by ^1^H-NMR analysis; ^2^ determined by SEC using polystyrene standards.

**Table 2:** Properties of the bio-polyols ^1^.

Sample ^1^	Composition ^2^	Viscosity ^3^ (Pa·s)	*M*_n_ ^4^ (g/mol)	Ð ^4^	Acid Number ^5^ (mg KOH/g)	OH Number ^6^ (mg KOH/g)	OH Functionality ^7^
BPO 1	ELO, 3PBA	470.3	2079	1.2	15	88	3.3
BPO 2	ESO, 3PBA	24.26	1869	1.31	20	93	3.1
BPO 3	ELO, CA	4.70	1769	1.36	3	67	2.1
BPO 4	ESO, CA	3.03	1797	1.15	2	87	2.8
BPO 5	p-ESO, CA	0.49	1546	1.16	5	67	2.0

^1^ Reaction conditions: for BPO 1–4 see Ref. [7]; for BPO 5, epoxy/carboxyl ratio = 1:1, T = 130 °C, t = 16 h; ^2^ ESO = epoxidized soybean oil, CA = caprylic acid, ELO = epoxidized linseed oil, 3PBA = 3-phenyl butyric acid; ^3^ measured at 20 °C; ^4^ determined by SEC using polystyrene standards; ^5^ determined according to IUPAC 2.201 standard method; ^6^ determined according to ASTM D 4274; ^7^ calculated by the formula: *M*_n_ × OH_number_/(56.1 × 1000).

**Table 3:** ^1^H and ^13^C NMR chemical shifts of prepolymer from BPO 4 aromatic moiety of the most abundant species.

Structure	Scheme 4	Proton	ppm	Carbon	ppm
2,4	2, 3, 4	Hc, Hc′, Hc″	7.93	Cc	110.3
	1	Hc‴	7.83	Cc‴	112.9
	2, 4	Hb, Hb″	7.19–7.11	Cb	113.6
	1, 2, 3, 4	Ha‴, Ha, Ha′, Ha″	7.12	Ca	130.3
	3	Hb’	6.49	Cb’	n.a.
				C-N	136.7
	1	Hb‴	7.12	Cb‴	115.8
	1, 2, 3, 4	Hd, Hd′, Hd″	2.22	Cd	16.63
2,6	6, 7	Hb, Hb′	7.47	Cb	119.45
	5	Hb”	7.3		122.04
	5, 6, 7	Ha, Ha′, Ha″	7.20	Ca	126.1
	7			Ca’	119.45
	6	Hc	6.47	Cc	n.a.
	5, 6, 7	Hd, Hd′, Hd″	2.16	Cd	11.86

n.a. = not assigned.

**Table 4:** Formulation of the foams used in this study.

	TDI-1 TDI-2, TDI-3, TDI-4, TDI-5	TDI-1W	TDI-3.2P	TDI-ref	TDI-refW
Bio-polyol	44	44	67	0	0
PPG	56	56	33	100	100
Glycerol	10.6	13.0	11.0	10.6	12.5
Water	2.6	5.2	2.6	2.6	5.3
Silicone surfactant	3.9	4.4	3.9	3.8	4.4
DABCO	0.6	0.7	0.6	0.7	0.7
DBT	0.1	0.1	0.1	0.1	0.1
Isocyanate	62	99	65	64	93
Isocyanate index	100	100	100	100	100

Units are in parts per hundred parts (pphp).

**Table 5:** Cream time, free rise time, density, strut thickness and pore size of PU foams.

Sample	BPO Composition ^1^	Cream Time (s)	Free Rise Time (s)	Strut Thickness (µm)	Density (kg/m^3^)	Pore Size ^2^ (µm)
TDI-1	ELO/3PBA	10	25	17 ± 6	72 ± 4	68 ± 24
TDI-1W	ELO/3PBA	8	18	15 ± 7	44 ± 2	81 ± 28
TDI-2	ESO/3PBA	15	31	26 ± 12	90 ± 17	152 ± 50
TDI-3	ELO/CA	15	38	21 ± 5	84 ± 4	54 ± 24
TDI-3.2P	ELO/CA	18	44	27 ± 10	87 ± 5	124 ± 47
TDI-4	ESO/CA	14	39	14 ± 6	77 ± 2	124 ± 52
TDI-5	p-ESO/CA	20	41	20 ± 4	63 ± 6	257 ± 60
TDI-ref	-	20	45	36 ± 10	74 ± 6	104 ± 48
TDI-refW	-	10	23	22 ± 6	42 ± 2	300 ± 97

^1^ ESO = epoxidized soybean oil, CA = caprylic acid, ELO = epoxidized linseed oil, 3PBA = 3-phenyl butyric acid, ^2^ pore size determined as $circle\;equivalent\;diameter=\sqrt{\mathrm{Area}/\pi }$.

**Table 6:** Thermal and mechanical properties of PU foams.

Sample	Composition	*T*_5%_ (°C)	*T*_max,1_ (°C)	*T*_max,2_ (°C)	*E* (kPa)	*CDV* (kPa)
TDI-1	ELO-3PBA	244	269	419	484 ± 3	56 ± 2
TDI-1W	ELO-3PBA_2.5%water	237	278	411	171 ± 8	23 ± 3
TDI-2	ESO-3PBA	203	296	407	330 ± 64	33 ± 2
TDI-3	ELO-CA	266	302	418	257 ± 36	30 ± 7
TDI-3.2P	ELO-CA	253	283	410	112 ± 9	14 ± 2
TDI-4	ESO-CA	240	275	408	369 ± 56	36 ± 7
TDI-5	p_ESO-CA	252	284	410	260 ± 24	26 ± 3
TDI-ref	PPG	250	278	387	127 ± 15	22± 2
TDI-refW	PPG_2.5%water	246	290	393	142 ± 30	15 ± 1

## Data Availability

The data presented in this study are available on request from the corresponding author.

## Supplementary Materials

The following supporting information can be downloaded at: https://www.mdpi.com/article/10.3390/polym15224423/s1, Figure S1: The 1H-NMR spectrum of ELO; Figure S2: FT-IR spectrum of TDI-ref foam; Figure S3: FT-IR spectrum of TDI-refW foam; Figure S4: FT-IR spectrum of TDI-1 foam; Figure S5: FT-IR spectrum of TDI-1W foam; Figure S6: FT-IR spectrum of TDI-5 foam; Figure S7: SEM micrograph at low magnification of TDI-1W foam; Figure S8: SEM micrograph at low magnification of TDI-3 foam.
