# Supplementary material for: Flexible Polyurethane Foams from Bio-Based Polyols: Prepolymer Synthesis and Characterization
Source: Polymers (Basel). 2023 Nov 16;15(22):4423. doi: 10.3390/polym15224423 (PMC10675359; doi:10.3390/polym15224423)
Supplement: Supplementary file 1 [file polymers-15-04423-s001.zip › polymers-2682513-supplementary.pdf]

Flexible Polyurethane Foams from Bio-Based Polyols: prepolymer synthesis and characterization

Simona Losio*, Angelica Cifarelli, Adriano Vignali, Simona Tomaselli, Fabio Bertini

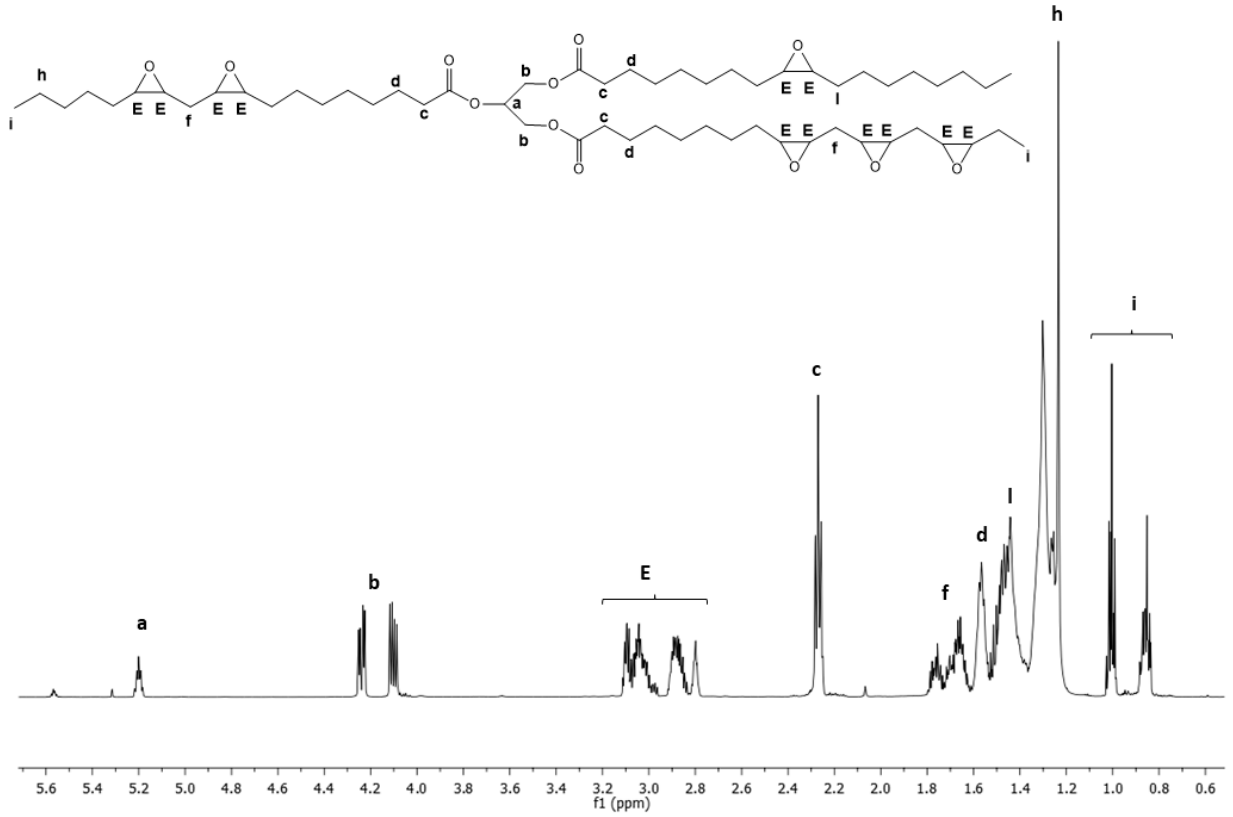

Figure S1. ^{1}H-NMR spectrum of ELO.

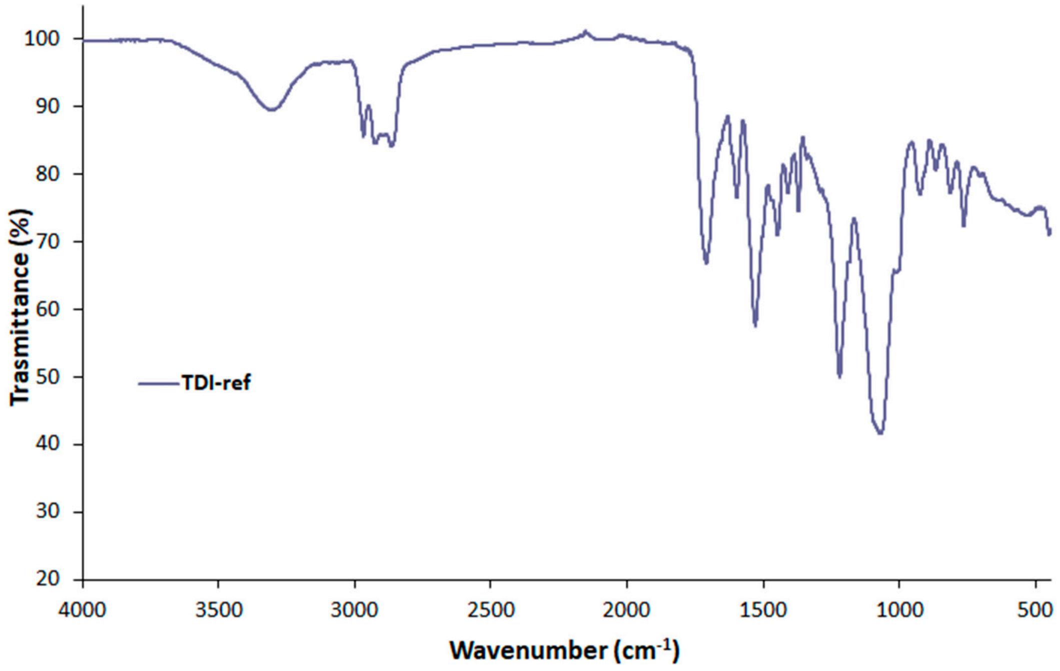

Figure S2. FT-IR spectrum of TDI-ref foam.

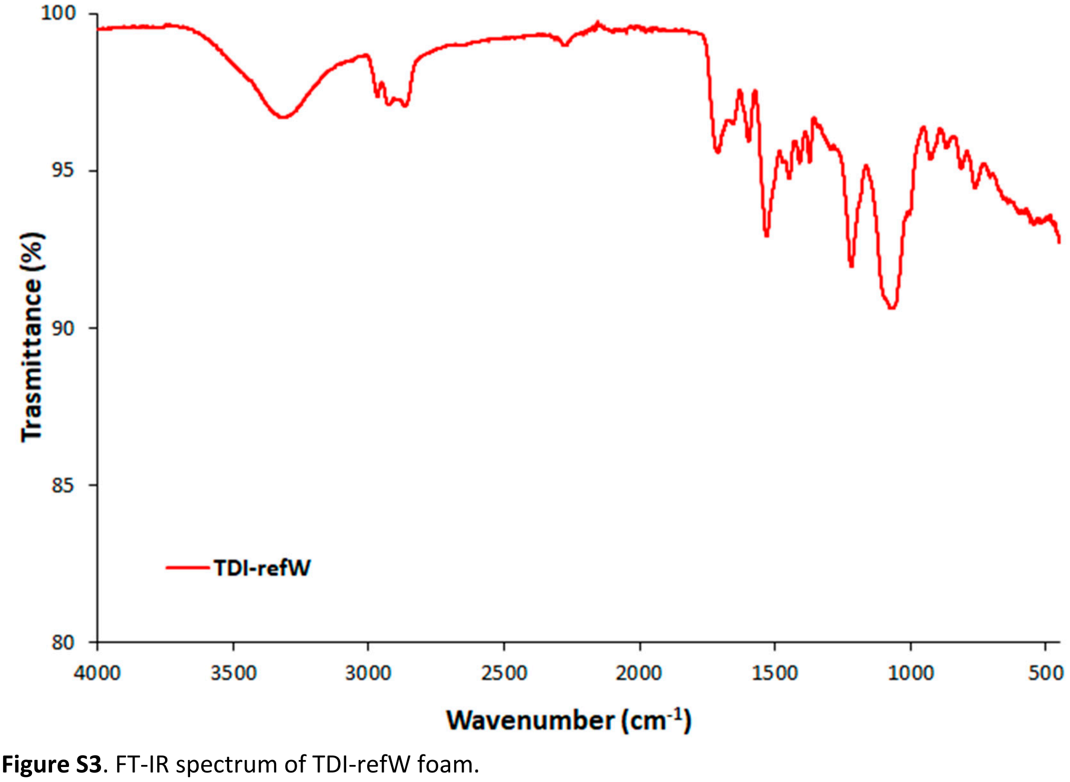

Figure S3. FT-IR spectrum of TDI-refW foam.

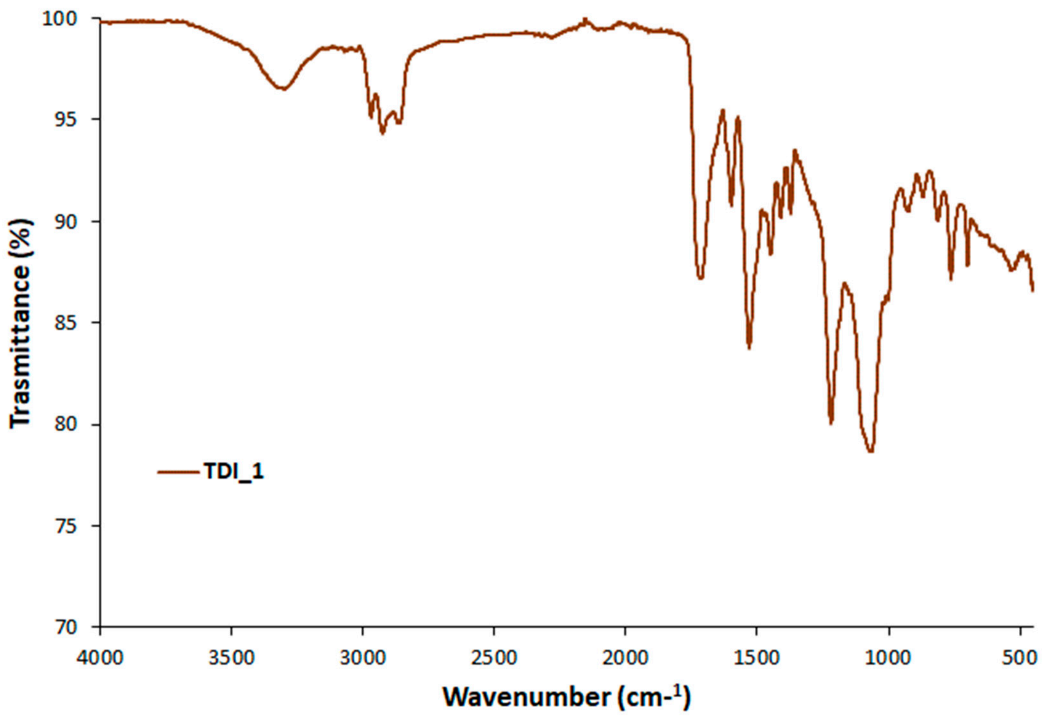

Figure S4. FT-IR spectrum of TDI-1 foam.

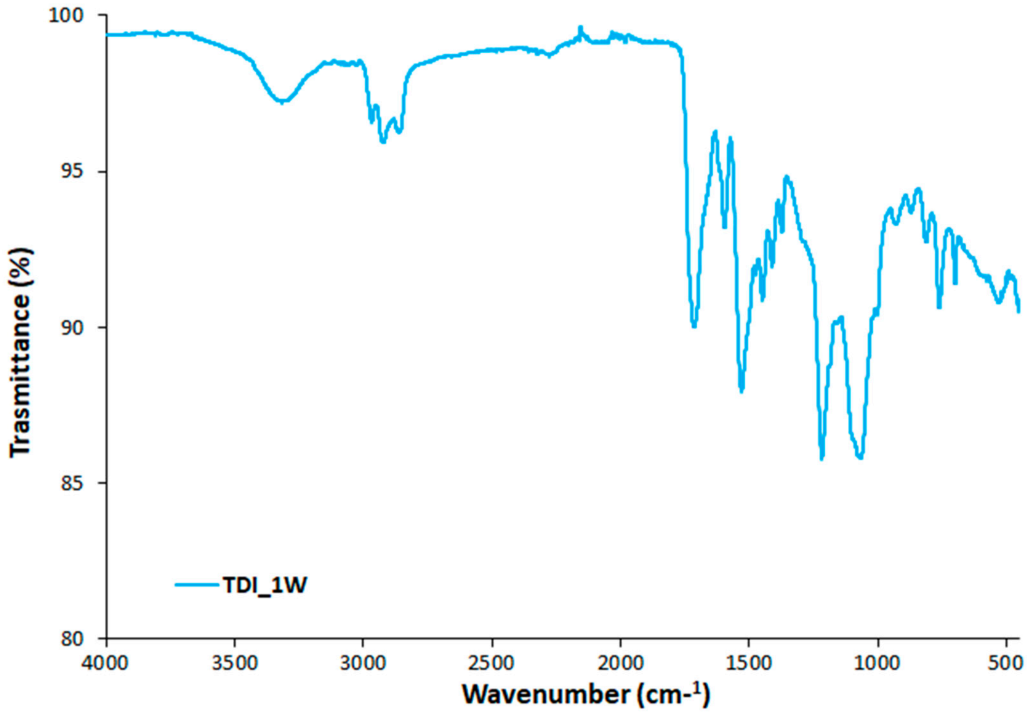

Figure S5. FT-IR spectrum of TDI-1W foam.

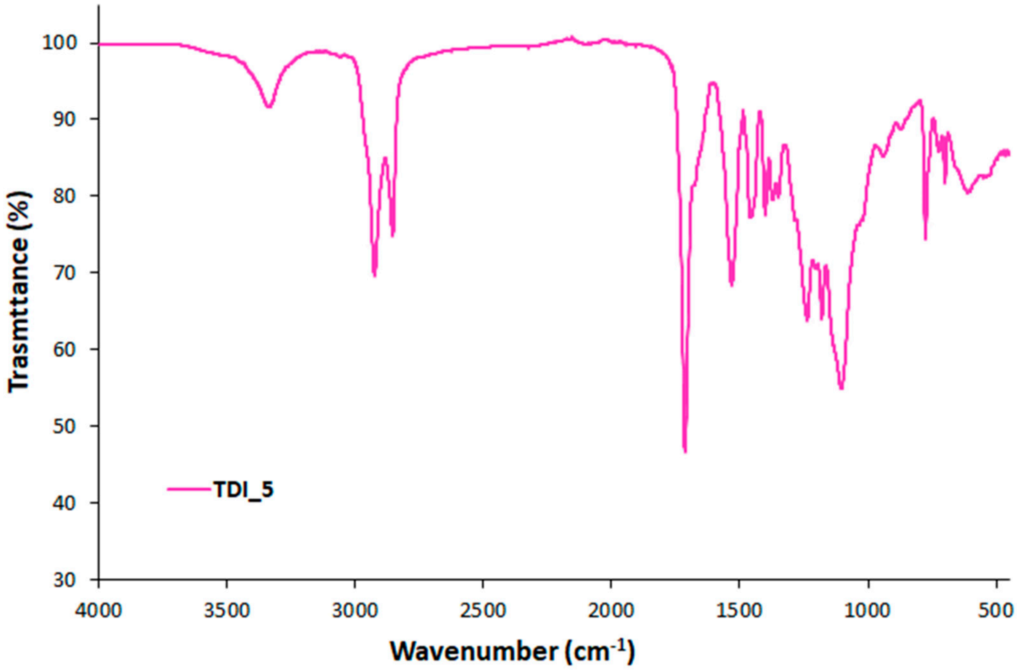

Figure S6. FT-IR spectrum of TDI-5 foam.

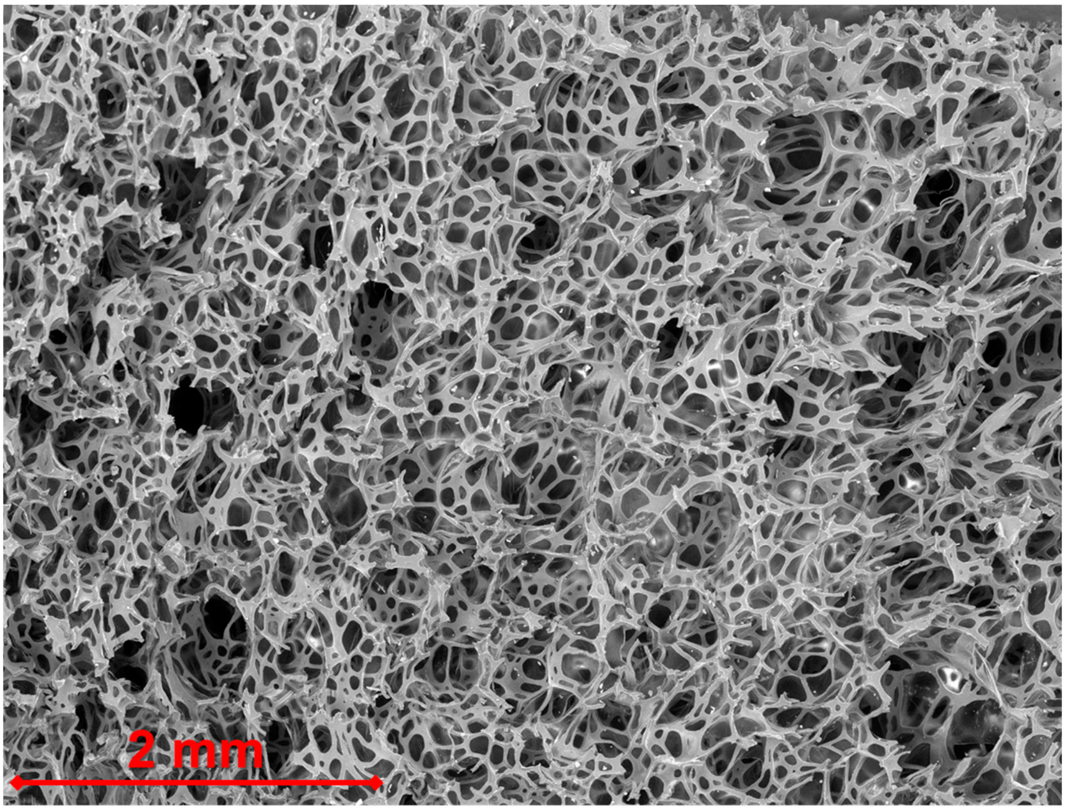

Figure S7. SEM micrograph at low magnification of TDI-1W foam.

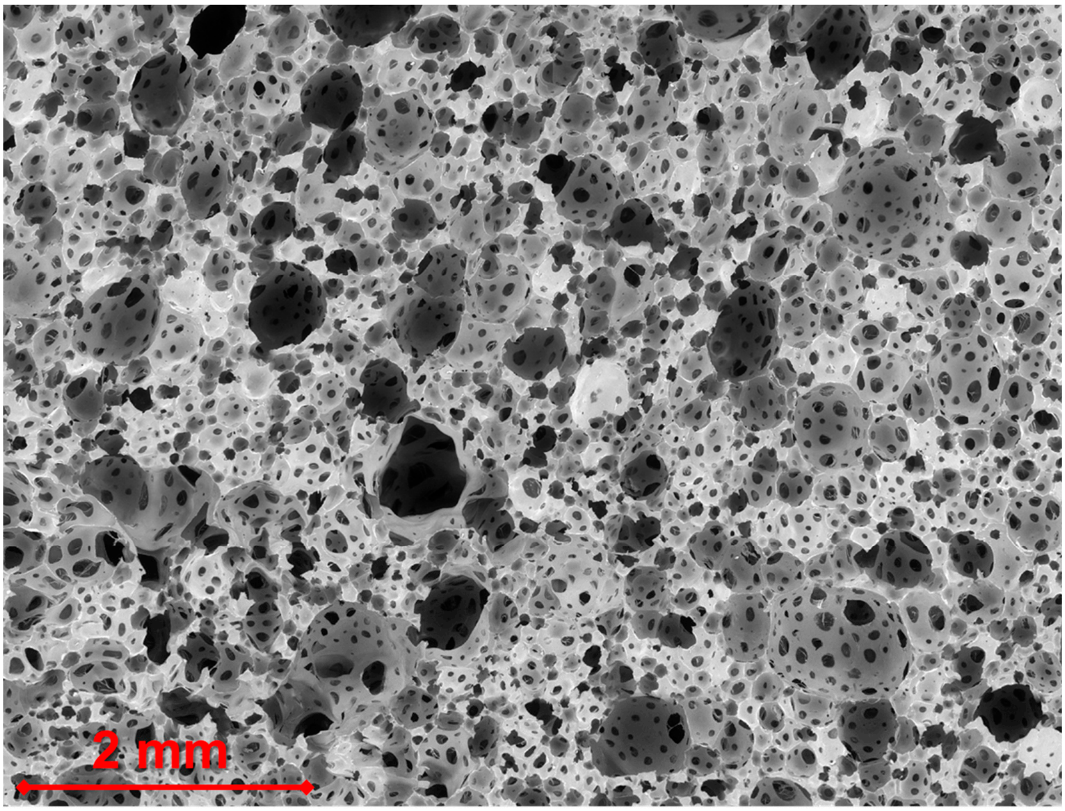

Figure S8. SEM micrograph at low magnification of TDI-3 foam.
